# Annotations

**Published:** 1896-12-26

**Authors:** 


### Annotations.

### The Election to the General Medical Council.

It seems clear that the recent election of direct representatives to the General Medical Council was to a large extent decided on the midwives' question, and so far as a poll of 54 per cent. of the profession in England can be taken to mean anything, the vote given goes against registration. We need hardly point out how greatly such a vote tends to weaken the influence of the medical profession in regard to any legislation on the subject which may take place. Both the General Medical Council and the British Medical Association have already given their sanction to proposals involving the registration of midwives, and the votes given to Dr. Rentoul and Mr. Brown, who are both absolutely opposed to these proposals, go in the opposite direction, and merely indicate how little unanimity there is in the profession upon the subject. All the three bills which have been brought forward, that introduced by Lord Balfour, that drawn up by a sub-committee of the British Medical Association, and that proposed by the Lancashire and Cheshire branch of the same association, sin against the creed of the newly-appointed members of the General Medical Council in that they institute a register and give legal sanction to the practice of midwifery by those who obtain access to it. It will be interesting then to watch the action of these gentlemen. Will they merely oppose the suggestions of others or will they put forward some plan of their own? They will probably be content to do the former and will refrain from putting their fingers into the fire. But it cannot be long before something is done to protect the women of England from the vagaries of the uneducated midwife; the question of interest is, What will be done? Will the law-makers of this country register the midwife and make her independent, or can they be so persuaded of the good faith of the medical profession, and of its sympathy with the general desire to secure the proper education of the midwife, as to be tempted to place her under medical control in the performance of her daily work? It is here that the recent election will tell against a reasonable settlement, for the politicians who will in the end have to decide this matter can hardly avoid coming to the conclusion that the votes then given express a feeling in the medical profession hostile to the midwife, and this is certainly not likely to increase their readiness to hand her over to the enemy.

### The Air of Bedrooms.

When there is too much water in the atmosphere the person who breathes it is to a certain limited extent deprived of his due supply of oxygen, and an elementary beginning of suffocation is perceived in his chest. Most middle-aged and all old persons have felt this; and all persons also who have weak hearts or impaired lungs. Now the air of bedrooms is exceedingly liable to be overcharged with watery vapour. The most obvious reasons for this are that many bedrooms are never warmed with fires, and that their windows are often left open all day until dusk, and sometimes even to the very hour of going to bed. Let us think of some of the consequences of going to bed in very damp air. A delicate or an aged person leaves a warm drawing-room, say, at half-past eleven, a drawing-room in which there was a temperature of 68 degrees; he enters a cold, damp bedroom, say, at a temperature of 38 degrees. The air in the drawing-room was dry, perhaps a little too dry. The air in the bedroom is saturated with cold watery vapour. The person we are thinking of, so soon as he enters the bedroom, chokes and gasps and coughs for half an hour at least, and sometimes brings on such an attack of asthma, or, as he calls it, "stuffiness" of the chest, that he can hardly breathe at all. He may even lose his night's sleep, and be ill for some days after such an exposure. Now, common sense says, "Make an effort to bring the atmosphere of the bedroom nearer in point both of dryness and warmth to the atmosphere of the drawing-room; and then not only will a man feel as comfortable in the bedroom as in the drawing-room, but even more comfortable. He will neither gasp, nor choke, nor cough, but will go to sleep with ease and comfort. Common sense teaches some people all this. But to those who have no special regard for common sense science tells the same tale, and she speaks with a voice whose authority not even the most learned will question.

### Coroners and Juries.

A matter of considerable public interest was recently brought before the Lord Chancellor by a deputation from the Control Department of the London County Council, urging certain reforms in the law as to coroners' inquests. The reforms proposed by the deputation were mainly in the directions of lessening the number of jurymen required at each inquest, and of diminishing the number of inquests by appointing special medical officers to investigate the cause of death. Lord Halsbury stated that he saw many advantages in the proposal to create medical investigators to make the preliminary investigations, but he thought that grave difficulties might be experienced in any attempt to reduce the number of the jury. The latter is a point we will not urge. The British jury is to the legal mind the ultimate arbiter of all matters of fact, and it is not surprising to find that a Lord Chancellor hesitates before tampering with so time-honoured a well of truth But as to the necessity of instituting some kind of official investigation into the cause of death in all cases in which no medical certificate is produced there can be no manner of doubt. In the thirty-three large towns as to which the Registrar-General issues weekly returns over 3,000 persons a year are buried without any medical certificate, and in the country districts the proportion is probably considerably greater. To throw all these cases on the coroner and his jury would be quite out of the question, and, moreover, would lead to nothing so long as coroners maintain their present attitude in regard to post-mortem examinations. To summon a jury and drag twelve or fourteen men from their work that they may go through the farce of listening to all sorts of irrelevant evidence, and then to tell them that if they insist upon having a post-mortem examination, on which alone they can found a reasonable verdict, they must come again and sacrifice another day's work, is a piece of cruel tyranny in which coroners are only too fond of indulging at the expense of unfortunate jurymen. Nominally, this is done to save expense; but there is no economy in futile inquests, and coroners apparently forget that they themselves, their officers, their courts, their juries, and especially their verdicts, are worthless, and even worse, as giving a cloak to crime, unless the verdicts are founded upon proper evidence.

